# Relative adrenal insufficiency in burns

**DOI:** 10.1186/cc12383

**Published:** 2013-03-19

**Authors:** A Mokline, L Gharsallah, I Rahmani, H Oueslati, B Gasri, S Tlaili, R Hammouda, A Ksontini, A Ghanem, AA Messadi

**Affiliations:** 1 Burn and Trauma Center, Tunis, Tunisia

## Introduction

Relative adrenal insufficiency (RAI) is an uncommon disorder among burn patients, which can often go unrecognized. However, there are no or almost no data about the incidence of this disease in burns. The goal of the current study is to evaluate the incidence of RAI in burn patients during the acute phase.

## Methods

A prospective study, approved by our Institutional Ethics Committee, was conducted in a 20-bed adult burn ICU at a university-affiliated teaching hospital in Tunis. Patients admitted within the first 24 hours post burn with greater than 10% total body surface area (TBSA) burned were enrolled in this study from 1 January 2009 to 30 June 2010. Exclusion criteria were pregnancy, history of adrenal insufficiency, or steroid therapy within 6 months prior to burns. A short corticotrophin test (250 g) was performed, and cortisol levels were measured at baseline (CS T0) and 60 minutes post test. Adrenal insufficiency was defined by a response ≤9 g/dl. Relative adrenal insufficiency was further defined by a baseline cortisol >20 g/dl.

## Results

Patients were assigned into two groups: G1 (RAI, *n *= 7) and G2 (absence AI, *n *= 11). Comparative study of the two groups shows the results presented in Table 1.

**Table 1:** Characteristics of the two groups

	G1	G2	*P *value
Age	38 ± 13	34 ± 12	NS
TBSA	57.8 ± 30	26 ± 22	0.019
UBS	158 ± 100	70 ± 99	NS
ABSI	8 ± 3	6 ± 3	NS
CS T0	37 ± 11	19 ± 14	0.01
D CS	2.2 ± 1.8	19 ± 6	<0.001
Shock	6 (86%)	4 (36%)	0.04

## Conclusion

RAI is common in severely burned patients during the acute phase, and is associated with shock. Further prospective controlled studies will be necessary to establish risk factors of RAI in severely burned patients and its impact on their prognosis.

